# Occlusal contact area changes with different retention protocols using T-Scan III: An observational study

**DOI:** 10.21142/2523-2754-1303-2025-251

**Published:** 2025-08-31

**Authors:** Arezoo Jahanbin, Haniyeh Ghorbaninezhad, Mostafa Abtahi, Farnaz Zia, Ziba Shirkhani

**Affiliations:** 1 Department of Orthodontics, School of Dentistry, Mashhad University of Medical Sciences. Mashhad, Iran. jahanbinA@mums.ac.ir, abtahim@mums.ac.ir Mashhad University of Medical Sciences Department of Orthodontics School of Dentistry Mashhad University of Medical Sciences Mashhad Iran jahanbinA@mums.ac.ir abtahim@mums.ac.ir; 2 General Dentist, School of Dentistry, Mashhad University of Medical Sciences. Mashhad, Iran. haniyeh.gh@gmail.com Mashhad University of Medical Sciences General Dentist School of Dentistry Mashhad University of Medical Sciences Mashhad Iran haniyeh.gh@gmail.com; 3 Department of Orthodontics, School of Dentistry, Guilan University of Medical Sciences. Rasht, Iran. farnaz_ziya91@yahoo.com Gilan University of Medical Sciences Department of Orthodontics School of Dentistry Guilan University of Medical Sciences Rasht Iran farnaz_ziya91@yahoo.com; 4 Statistician, Dental Research Center, Mashhad University of Medical Sciences. Mashhad, Iran. shirkhaniz2@mums.ac.ir Mashhad University of Medical Sciences Dental Research Center Mashhad University of Medical Sciences Mashhad Iran shirkhaniz2@mums.ac.ir

**Keywords:** retention, occlusion, essix retainer, T-Scan III, retención, oclusión, retenedor Essix, T-Scan III

## Abstract

**Introduction:**

The retention phase in orthodontics is essential for maintaining corrected occlusion and preventing relapse. Clear retainers like Essix are popular for their aesthetics and affordability, but their occlusal coverage might impact anterior and posterior contacts. Modified versions, such as Occless Essix, have been introduced to address these concerns. The aim of this study was to compare occlusal changes over time between Essix and Occless Essix retainers using the T-scan III system.

**Materials and Methods:**

This analytical observational study involved 30 orthodontic patients treated with MBT 0.022-inch brackets and first premolar extractions. Patients were divided into two groups: one with Essix retainers and the other with Occless Essix retainers. Occlusal contacts and their intensities in anterior and posterior regions were recorded immediately after debonding (T0) and three months later (T1) using the T-scan III system. Statistical analysis used Mann-Whitney, T-test, and Chi-square tests, with significance set at 5%.

**Results:**

After three months, the Occless Essix group showed increased posterior contact intensity and a slight decrease in anterior contact intensity. Conversely, the Essix group had increased anterior contact intensity and decreased posterior contact intensity. These changes were not statistically significant (P=0.17). The Occless Essix group had an increased number of posterior occlusal contacts and a decreased number of anterior contacts, while the Essix group showed a slight increase in anterior contacts. These changes were not statistically significant (P<0.05).

**Conclusion:**

There were no significant differences between Essix and Occless Essix retainers regarding changes in occlusal force intensities or the number of occlusal contacts after three months.

## RESUMEN

**Introducción:**

La fase de retención en ortodoncia es esencial para mantener una oclusión corregida y prevenir la recidiva. Los retenedores transparentes, como Essix, son populares por su estética y precio accesible, pero su cobertura oclusal podría afectar los contactos anteriores y posteriores. Se han introducido versiones modificadas, como Occless Essix, para abordar estas preocupaciones. El objetivo del presente estudio fue comparar los cambios oclusales a lo largo del tiempo entre los retenedores Essix y Occless Essix utilizando el sistema T-Scan III.

**Materiales y métodos:**

Este estudio observacional analítico incluyó a 30 pacientes de ortodoncia tratados con *brackets* MBT de 0,022 pulgadas y con extracciones de primeros premolares. Los pacientes se dividieron en dos grupos: uno con retenedores Essix y el otro con retenedores Occless Essix. Los contactos oclusales y sus intensidades en las regiones anterior y posterior se registraron inmediatamente después del descementado (T0) y tres meses después (T1), utilizando el sistema T-Scan III. El análisis estadístico utilizó las pruebas de Mann-Whitney, T de Student y chi-cuadrado, con una significancia del 5%.

**Resultados:**

Después de tres meses, el grupo Occless Essix mostró un aumento en la intensidad de contacto posterior y una ligera disminución en la intensidad de contacto anterior. Por el contrario, el grupo Essix mostró un aumento en la intensidad de contacto anterior y una disminución en la intensidad de contacto posterior. Estos cambios no fueron estadísticamente significativos (p = 0,17). El grupo Occless Essix presentó un aumento en el número de contactos oclusales posteriores y una disminución en el número de contactos anteriores, mientras que el grupo Essix mostró un ligero aumento en los contactos anteriores. Estos cambios no fueron estadísticamente significativos (p < 0,05).

**Conclusión:**

No se observaron diferencias significativas entre los retenedores Essix y Occless Essix en cuanto a los cambios en la intensidad de la fuerza oclusal ni en el número de contactos oclusales después de tres meses.

## INTRODUCTION

One of the main challenges after orthodontic treatment is the maintenance of the correct position of the teeth. Relapse refers to the undesirable changes and movements of the teeth after orthodontic treatment, which cause the aligned teeth to return to their initial position. ^1^

The primary goal of orthodontic treatment is to achieve an ideal tooth relationship. The most important factor in the stability of occlusion is the presence of occlusal contacts on the functional cusps. ^(2, 3)^ "Settling" refers to the vertical movement and displacement of posterior teeth after orthodontic treatment, which is a beneficial type of relapse and increases the number of occlusal contacts. The best retainer is one that has the least amount of relapse and optimal settling. ^3^^-^^5^

The Essix retainers were first introduced in 1993 by Sheridan et al, and have become an increasingly popular option due to improved aesthetics, ease of fabrication and reduced cost. They are available in various forms, covering all teeth, from canine to canine, and from premolar to premolar. ^6^ )This retainer is made of clear plastic and lacks clasps, making it more aesthetically pleasing and cost-effective compared to the Hawley retainer.It is quicker and easier to manufacture and has suitable durability. Essix also performs better in maintaining the position of anterior teeth. ^(7, 8)^

Various methods have been used to quantitatively examine occlusion, such as alginate impression materials, silicone putty, articulating paper, silk strips, Shimstock film, occlusal spray, and occlusal ultrasound. ^9^ Given that many studies have proven the inability of the above systems to accurately display occlusal status, the use of digital systems like T-scan III will be effective. 

The aesthetic appeal of clear retainers, such as Essix, has increased their popularity. However, concerns about their full occlusal coverage affecting occlusal contacts over time have emerged. The Occless Essix retainer, a modified version of the Essix, was developed to address these concerns by leaving the posterior occlusal surfaces uncovered. This design aims to allow for more natural occlusal contact and settling during the retention phase. Despite its growing clinical use, there is limited research comparing the occlusal outcomes of traditional Essix versus Occless Essix retainers. This study aims to evaluate and compare anterior and posterior occlusal changes using T-scan III technology over an initial 3-month retention period.

## MATERIALS AND METHOD

### Selection of Participants

This is an observational study, and no interventions were assigned by the researchers. The institutional Ethics Committee granted ethical clearance (IR.MUMS.DRC.REC.1401.161dated April-15-2022). Verbal and written informed consent were obtained from the patients and parents (in the case of patients under 18 years old) before inclusion in the study. The samples for this investigation consisted of patients who had completed fixed orthodontic treatment at the Department of Orthodontics, School of Dentistry, Mashhad University of Medical Sciences, Mashhad, Iran, and the Special Clinic of the Faculty and were scheduled to have their orthodontic appliances removed. 

### Inclusion Criteria:

• Age between 15 to 28 years at the end of treatment
• Skeletal Class I relationship
• Completeness of the dental system before the start of treatment
• Treated with fixed orthodontic appliances (MBT 22), along with the extraction of 4 first premolars in both arches
• Normal occlusion at the end of treatment with desirable overjet and overbite (2-3 mm)

### Exclusion Criteria:

• Individuals with parafunctional habits, TMJ issues, muscle spasms, and specific anomalies
• Periodontal issues at the end of treatment
• Continuous use of medication, especially psychiatric drugs
• Ortho-surgical patients or those with cleft lip and palate
• Non-cooperative patients during the treatment
• Hypodontia requiring tooth replacement in the retainer

The sample size for each group with 95% confidence and 80% power, was calculated as n=12, according to data obtained from a previous study ^3^ where the mean ± standard deviation of the changes in occlusal contacts in the control group (Essix) was 3.13 ± 1.6 and that of the intervention group (Occless Essix in this study) was 5.72 ± 2.6. assuming an alpha significance level of 0.05. Considering an approximate dropout rate of 10%, a total of 15 individuals per group, amounting to 30 in total, were included in the study. This sample size was calculated using G*Power software with an effect size of 1.23. Table 1 presents the demographic characteristics of the participants. The enrollment started in April 2024 and was completed by July 2024.

**Table 1:** Comparison of baseline characteristics in the study groups [the quantitative variables have been shown by mean ± SD and qualitative variables by number (%)]

		Essix	Occless Essix	p-value
Sex	Female	9	9	1.00
	Male	6	6	
	Total	15	15	
Age		19.83±3.01	19.83±3.91	1.00

A number of orthodontic patients aged 15-30 years who were ready for debonding and were undergoing fixed treatment with the MBT bracket system 0.022 inch and had first premolar extractions in both jaws were initially selected. If the patient met all the inclusion criteria for the study, an informed consent document was taken from patients or their parents/legal guardians after a brief explanation of the treatment process and upon their agreement, the patients were included in the current study.

**Figure 1:**
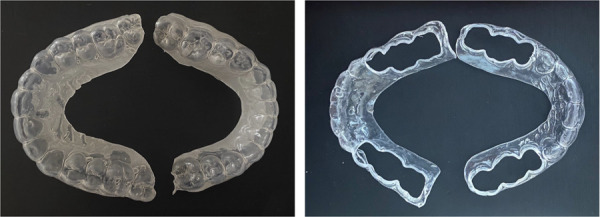
Essix and Occless Essix

30 orthodontic patients, comprising 18 women and 12 men, were included in the study and were divided into two equal groups: Essix or Occless Essix (Figure 1), in a non-random manner. The patients were selected from different centers, and the division was based on the orders and diagnoses of the treating orthodontist. At the debond appointment after removing the appliances, alginate impressions (Chromogel, Iran) were taken from the upper and lower arches. Working casts of both arches were immediately obtained from the impressions, and the retainers were fabricated by a laboratory technician. The retainers were constructed using clear polyvinyl siloxane sheet (Forplast thermoforming tiles, Roko) with a thickness of 1.5 mm, covering 1-2 mm on the buccal gum surface and 3-4 mm on the palatal gum surface. The retainers were delivered to the patients within 48 hours of debonding, and the patients were given instructions on how to use and care for the retainers. For the Occless Essix, a basic Essix was initially created, and then the occlusal surfaces of the posterior teeth were opened using a bur. The participants included in two groups:

Group 1 (Essix retainer): The patients in group 1 (15 patients) received Essix retainers in both jaws, which cover the occlusal surfaces of the teeth as well. Patients were instructed to wear this appliance full-time for 3 months except for eating and oral hygiene care and then part-time (for 12 hours a day) for 9 months.

Group 2 (Occless Essix retainer): In this group, 15 participants received Occless Essix retainers for upper and lower jaws. The patients were instructed to wear the Occless Essix retainer 3 months full-time except for eating and oral hygiene care and then part-time (for 12 hours a day) for 9 months.

**Figure 2:**
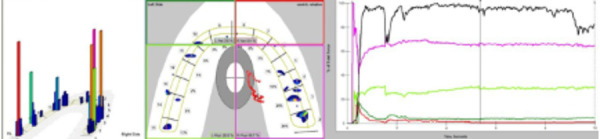
Two and three-dimensional graph of T-scan III

The T-scan III device (Tekscan, Inc., S. Boston, MA, USA) which is digital occlusal analysis system, was used in this study. This method utilized a polyester sheet with a thickness of 85 microns, containing 2500 pressure-sensitive cells, which transmitted the applied pressure data to the software. The data is recorded in two-dimensional and three-dimensional formats within the software, allowing for the identification and recording of occlusal contact locations, the number of contacts, and the force distribution. The software effectively demonstrates the distribution of occlusal force across the entire dental arch. The duration from the first contact to the Maximum Intercuspation (MIP), as well as the path of jaw movement during mouth closure, can also be recorded and visualized.(7, 10)The graphs generated in the software feature an x-axis representing time in seconds and a y-axis indicating the intensity of the recorded force as a percentage of the maximum biting force (Figure 2).

### Data collection and measurements:

#### Analysis using T-scan III

All T-scan III recordings were performed by a single trained operator. Prior to data collection, the operator underwent standardized training, and calibration was carried out according to the manufacturer’s instructions to ensure accuracy and intra-operator consistency.

For each use of the T-scan III, patients were seated upright in a dental unit, and their Frankfurt plane was aligned parallel to the floor. The patients' lower jaw was manipulated into Centric Relation (CR) in a physiological position^11^ by asking the patients to place their tongue against the back of their palate and swallow their saliva.

Patients were instructed to bite the sensor, which was adjusted so that its anterior part rested on the midsagittal line of the sheet and between the two central incisors. Subsequently, patients firmly occluded three consecutive times in Maximum Intercuspation (MIP). To avoid muscle fatigue errors, patients were given a 3-minute rest between each bite.

The following occlusal parameters were evaluated from the recorded data:

• Anterior force distribution
• Posterior force distribution
• The number of anterior tooth contacts
• The number of posterior tooth contacts

It is important to note that since patients were assessed three times consecutively by T-scan at T1 and T0, varying values of occlusal contact intensity were obtained; thus, the mean contact intensity was calculated and provided to the statistician. 

### Statistics

The normal distribution of the data was confirmed by the Shapiro-Wilk test (P>0.05). A t-test was used to examine the effect of age between the two groups. The analysis of the gender variable was conducted using the Chi-square test. For comparing two independent groups with non-normal distribution, the Mann-Whitney test was used. For two groups with normal distribution, the t-test was applied again. The statistical analysis was performed using Statistical Package for Social Sciences (SPSS; version 21), and p-values less than 0.05 were considered statistically significant.

## RESULT

The study included 30 patients, 18 women (60%) and 12 men (40%), with an average age of 19.4 ± 3.8 years. The age and gender distribution of the subjects in the study groups were comparable (Table 1). No significant difference was found between the groups.

Force distribution: Table 2 demonstrates the mean and standard deviation (SD) of force distribution in two groups over the period of the experiment. The normality of the variables was first examined using the Shapiro-Wilk test. As shown in Table 2, there was no significant difference in force distribution between the two groups in the posterior and anterior regions at T0 (P = 0.3). Although the force distribution at the start of the investigation in the posterior region of the Essix group showed a higher value, this difference was not statistically significant. As indicated in Table 2 and based on the Mann-Whitney analysis, the force distribution in the posterior and anterior areas at T1 showed no significant difference between the two groups (P = 0.66).

**Table 2:** The mean and standard deviation (SD) of anterior force distribution in the study groups at the debond appointment (T0) and at 3 months later (T1).

	Study group	T0			T1		T1-T0	
		Mean	SD	Mean	SD	Mean	SD
	Essix	25.53	23.28	26.73	18.24	1.2	11.56
Anterior	Occless Essix	40.60	33.63	28.72	30.57	-11.88	34.24
	Statistical significance (p)	0.3			0.66		0.17	

In Table 2, the changes in the force distribution on posterior and anterior teeth over a three-month period were compared between two groups using a T-test. In the Essix group, the force distribution in the posterior region showed an increase. Additionally, the force distribution in the Essix group also showed a slight increase, while in the Occless Essix group, a decrease was observed after three months. These changes were not statistically significant (P = 0.17). 

The number of occlusal contacts: As shown in Table 3, at both times T0 and T1, the mean number of posterior occlusal contacts in the Essix group was slightly greater than in the Occless Essix group, while the mean number of contacts on the anterior teeth at T0 in both groups was nearly equal. The difference in the mean number of posterior and anterior occlusal contacts between the two groups based on the Mann-Whitney analysis at time T0 was not statistically significant (P = 0.77 for posterior and P = 0.83 for anterior). The mean number of posterior and anterior contacts in the Essix group was slightly greater than in the Occless Essix group. However, this difference in the mean number of anterior and posterior occlusal contacts between the two groups at time T1 was not statistically significant based on the T-test (P = 0.21 for posterior and P = 0.76 for anterior). The changes in the mean number of occlusal contacts were evaluated using a T-test. In both groups, there was a negligible increase in the number of contacts in the posterior region. Additionally, in the Essix group, the number of anterior contacts showed a slight increase, while in the Occless Essix group, a minor decrease was observed after three months. However, these changes in both the anterior and posterior regions were not statistically significant.

**Table 3:** Mean and standard deviation (SD) of posterior force distribution in the study groups at the debond appointment (T0) and 3 months later (T1).

	Study Group	T0		T1		T1-T0	
		Mean	SD	Mean	SD	Mean	SD
Posterior	Essix	74.46	23.28	73.26	18.24	-1.2	11.56
	Occless Essix	39.53	33.63	71.27	30.57	11.28	34.24
	Statistical Significance (p)	0.3		0.66		0.17	

**Table 4:** The mean and standard deviation (SD) of the number of anterior occlusal contact in the study groups at the debond appointment (T0) and 3 months later (T1).

	Study group	T0		T1		T1-T0	
		Mean	SD	Mean	SD	Mean	SD
Anterior	Essix	3.7	1.9	3.9	1.9	0.13	1.4
	Occless essix	3.7	2.3	2.9	2.1	-0.8	1.7
	Statistical significance (p)	0.83			0.21		0.8	

**Table 5:** The mean and standard deviation (SD) of the number of posterior occlusal contacts in the study groups at the debond appointment (T0) and 3 months later (T1).

	Study group	T0		T1		T1-T0	
		Mean	SD	Mean	SD	Mean	SD
Posterior	Essix	7.5	2.6	7.5	3.1	0	3.18
	Occless Essix	6.8	4.2	7.1	4.9	0.2	3.8
	Statistical Significance (p)	0.77			0.76		0.1	

## DISCUSSION

Prevention of unwanted changes after the completion of orthodontic treatment is one of the main challenges in orthodontics. ^12^^)^ The health of the TMJ and masticatory muscles is related to the occlusion achieved at the end of orthodontic treatment. Changes in occlusal contacts occur immediately after finishing orthodontic treatment as a result of settling post-treatment. Assessing contact points after orthodontic treatment is a good predictive factor for occlusal stability and preventing future relapse. The T-scan III device can display force values and the percentage distribution of force in a two-dimensional and three-dimensional manner, for each tooth and quadrant. Due to its high repeatability, speed, and ease of use chair-side, it is a good choice for assessing force distribution and the number of occlusal contacts compared to other methods. ^(3, 7)^ Wang et al. in 2011, ^13^ demonstrated that the T-scan III system shows high precision and repeatability in clinical conditions and is highly reliable in assessing occlusal contacts. Garcia et al. in 1997. ^14^^)^ found that saliva present in the mouth does not interfere with the T-scan III recording process and that this system retains high precision, sensitivity, and repeatability. 

An ideal retainer should be able to encompass the tooth from all directions while allowing for horizontal and vertical settling and preventing relapse. Clear retainers have been well-received by both patients and orthodontists due to their ease of fabrication, aesthetics, and minimal need for adjustments. However, the main drawback of these retainers is their occlusal coverage, which can lead to increased overbite and joint pain. Modifications to these clear retainers and the removal of occlusal coverage from the premolars and molars have been made to improve these mentioned disadvantages. The duration agreed upon by most orthodontists for the use of removable retainers is one year, which is based on the time needed for the reconstruction of PDL fibers and the collagen and elastic fibers of the gums. ^(3, 7)^

According to the results obtained, in the Occless Essix group, the force distribution of posterior contacts increased after three months. In the Essix group, the intensity of anterior contacts showed a slight increase, while in the Occless Essix group, a decrease was observed after three months; however, these changes were not significant. Conversely, in both groups, the number of posterior contacts showed a negligible increase after three months. Additionally, the Essix group showed a slight increase in the number of anterior contacts, while the Occless Essix group demonstrated a slight decrease after three months. Nevertheless, this amount of change was not significant. Given that the expected changes discussed at the beginning of the analysis occurred in this study, several possibilities arise. These include insufficient settling time, degrees of relapse resulting from the periodontal ligament (PDL) fibers of the posterior teeth immediately after treatment, and posterior dental intrusion due to occlusal coverage, similar to that of posterior bite blocks (although the thickness of the retainers is much less than that of bite blocks).

Ragunanthanan et al.,2022, (3) conducted a study using the T-scan III device and found that the lack of occlusal surface coverage of the posterior teeth with Occless Essix significantly increased occlusal contacts after six months of the retention period. The results of this study differ from the present research, which may be due to the six-month duration of Ragunanthanan's research; however, both studies reported a reduction in contacts in the anterior region. Omidkhoda et al.,2019, ^7^^)^ compared Hawley and Essix retainers using the T-scan III device. Similar to the present study, the changes in posterior and anterior contacts in the Essix group were not significant during the first three months. However, over a one-year period, they reported a significant increase in anterior contacts and a reduction in posterior contacts. Alkan and Kaya,2020, ^15^^)^ conducted a study using the T-scan III device and compared the distribution of occlusal forces and occlusal contact areas between three groups: Essix, Hawley, and fixed retainers at three and six months into the retention period. In the Essix group, changes in occlusal force intensity were not significant,similar to the current study. Additionally, changes in the occlusal contact area in the anterior region lacked significant statistical difference, and only in the posterior region did they show a significant increase over the six-month period. Aslan et al., 2013, ^16^^)^ compared the effects of Essix and Occless Essix on occlusal contacts. In the Occless Essix group, after nine months of use, a significant increase in posterior contacts and a decrease in anterior contacts were observed. These results were similar but not significant compared to the present study. In the Essix group, the increase in posterior contacts was similar to this study, and the decrease in anterior contacts in this group was significant. The major difference between this study and the present one was the method of assessing occlusal contacts, which was evaluated using silicone bites.

### Limitations

The limitations of this study were the small number of samples in each group and the short follow-up period. Additionally, the patients' usage of the retainers was assessed only through questioning, lacking adequate supervision. Considering that relapses occur over a longer duration and varies among individuals depending on various conditions, studies with a larger sample size and assessments over a longer time span would be more reliable. Also, other occlusal indices such as overbite and overjet were not evaluated in this study. Patients should be aware that despite the use of any retention appliance, relapse may still occur in some patients as a result of natural growth after the removal of orthodontic appliances. It is recommended that the choice between these two types of retainers be made considering factors such as clinical efficacy, periodontal tissue health, patient acceptance and comfort, and cost-effectiveness, which were not examined in this study. 

## CONCLUSION

Based on the conditions of this study:

• The number of contacts in both the anterior and posterior regions increased after 3 months of using Essix, but this increase was not significant.
• The use of Occless Essix retainers after three months reduced the number of contacts in the anterior region while increasing them in the posterior region; however, these changes were not significant.
• In the Occless Essix group, the force distribution in the posterior region increased after three months, while there was a slight decrease in the Essix group; however, this difference was minor and not significant.
• Overall, both types of retainers showed no difference in terms of force distribution and the number of occlusal contacts.
